# MicroRNA-212-5p Prevents Dopaminergic Neuron Death by Inhibiting SIRT2 in MPTP-Induced Mouse Model of Parkinson’s Disease

**DOI:** 10.3389/fnmol.2018.00381

**Published:** 2018-10-11

**Authors:** Sifan Sun, Xiaojuan Han, Xueting Li, Qiqi Song, Ming Lu, Miaomiao Jia, Jianhua Ding, Gang Hu

**Affiliations:** ^1^Department of Pharmacology, School of Medicine and Life Sciences, Nanjing University of Chinese Medicine, Nanjing, China; ^2^Affiliated Hospital of Nanjing University of Chinese Medicine, Nanjing, China; ^3^Department of Traditional Chinese Medicine, The Affiliated Drum Tower Hospital of Nanjing University Medical School, Nanjing, China; ^4^Jiangsu Key Laboratory of Neurodegeneration, Department of Pharmacology, Nanjing Medical University, Nanjing, China

**Keywords:** miR-212-5p, SIRT2, p53, autophagy, apoptosis, Parkinson’s disease

## Abstract

Recently, emerging evidences show that sirtuins (SIRTs) modulate aging progress and affect neurodegenerative diseases. For example, inhibition of SIRT2 has been recognized to exert neuroprotective effects in Parkinson’s disease (PD). However, current SIRT2 inhibitors are lack of selective property distinguished from its homolog. In this study, we found that SIRT2 protein level was highly increased in PD model, which was negatively regulated by miR-212-5p. In detail, miR-212-5p transfection reduced SIRT2 expression and inhibited SIRT2 activity. *In vivo* study, miR-212-5p treatment prevented dopaminergic neuron loss and DAT reduction by targeting SIRT2, which means miR-212-5p shows neuroprotective effect in PD. Mechanismly, we found nuclear acetylated p53 was up-regulation according to p53 is a major deacetylation substrate of SIRT2. Furthermore, decreased cytoplasmic p53 promoted autophagy in PD model, which was showed as autophagosomes, autophagic flux, LC3 B and p62 expression. Meanwhile, we also found miR-212-5p treatment somehow alleviated apoptosis in PD model, which might have some underlying mechanisms. In conclusions, our study provides a direct link between miR-212-5p and SIRT2-mediated p53-dependent programmed cell death in the pathogenesis of PD. These findings will give us an insight into the development of highly specifically SIRT2 inhibitor of opening up novel therapeutic avenues for PD.

## Introduction

Parkinson’s disease (PD) is one of the most common neurodegenerative diseases, which is characterized by the progressive loss of dopaminergic (DA) neurons in the substantia nigra compacta (SNc) and aggregation of Lewy bodies in neurons (Savitt et al., 2006). Although aging, genetic factors, oxidative damage, neuroinflammation, and programmed cell death (PCD) play vital roles in the pathogenesis of PD, the precise etiology remains unclear (Dexter and Jenner, 2013). Emerging evidences show that sirtuins (SIRTs) modulate the course of aging and affect neurodegenerative diseases (Jesko et al., 2017). It is well recognized that SIRT1 displays neuroprotective properties in PD (Herskovits and Guarente, 2014; Kida and Goligorsky, 2016). For instance, SIRT1 decreases α-synuclein-caused neuro toxicity by deacetylating HSP70 (Donmez et al., 2012) and inhibits activation of microglia by p53/caspase 3-dependent apoptosis (Ye et al., 2013). Unlike SIRT1 plays neuroprotective effects, SIRT2 aggravates the pathological damage induced by MPTP (Liu et al., 2014). In addition, inhibition of SIRT2 reduces DA neurons loss and blocks the α-synuclein toxicity (Outeiro et al., 2007). However, existing SIRT2 inhibitors, such as AK-1, AK-7, and AGK-2, are lacking of high specificity for SIRT2 over SIRT1, which restricts their pharmacological effects in PD therapy (Cui et al., 2014).

Seven SIRTs (SIRT1–7) are present in mammals. They target distinct protein substrates and are located in distinct subcellular compartments. SIRT2 not only is primarily cytosolic but also may enter the nucleus during the G2/M phase (Houtkooper et al., 2012). Thus, SIRT2 participates in the modulation of multiple and diverse biological processes, such as cell cycle, microtubule dynamics, metabolic networks and autophagy by deacetylating lysines on diverse proteins (Outeiro et al., 2007; Yu and Auwerx, 2009; Zhao et al., 2010; Beirowski et al., 2011; Maxwell et al., 2011). The tumor suppressor protein 53 (p53), one of the important substrates of SIRT2 (van Leeuwen et al., 2013), plays a dual role in autophagy according to its sub-location. Nuclear p53 induces autophagy, and cytoplasmic p53 acts instead as a repressor of autophagy (Levine and Abrams, 2008; Tasdemir et al., 2008). It is reported that SIRT2 inhibited lysosome-mediated autophagic turnover by interfering with aggresome formation in neuro cytotoxicity model (Gal et al., 2012). However, there is lack of study to explore the link between SIRT2 and p53-dependent autophagy in PD model. In addition, silence of SIRT1 decreases acetylation of p53 and inducing autophagy (Lee et al., 2015), which indicates that p53 is enigmatic in autophagy regulation. Thus, researchers are trying to develop highly selective drug-like inhibitors or agonists that show a unique mechanism (Rumpf et al., 2015). According to perturbation of micro RNAs (miRNAs) expression and specific targets in the pathophysiology of PD, miRNA-based therapy has been taken insights to PD treatment (Ma et al., 2013; Maciotta et al., 2013).

miRNAs are essential small RNA molecules (20–24 nt) that negatively regulate the expression of target genes at the post-transcriptional level. Substantial evidences suggest that miRNAs are critical in PD pathogenesis. For instance, miR-7 represses toxicity and cell death by targeting α-synuclein (Junn et al., 2009; Doxakis, 2010). In our previous work, we found miR-7 also enhances neurogenesis and inhibits neuroinflammation by targeting NLRP3 (Fan et al., 2016; Zhou et al., 2016). Studying miRNA involvement in PD might also could provide targets for innovative therapy. According to the development of miRNA-targeting oligonucleotides with improved pharmacological activity and optimized pharmacokinetic properties, miRNAs would be recognized as therapeutic agents against disease (Junn and Mouradian, 2012). Our preliminary data showed that miR-212-5p was decreased in PD model and might be a potential regulator of SIRT2 by bioinformatics prediction. In this study, we prepared MPTP-induced PD mice model *in vivo* and MPP^+^ stimulation *in vitro* with miR-212-5p gene therapy, so as to explore its functional and therapeutic role in PD model. We found SIRT2 expression in protein level remarkably increased without alteration in RNA level in the PD experimental model, inhibiting of SIRT2 by miR-212-5p could prevent DA neurons loss via promotes cytoplasmic p53-dependent autophagy. Meanwhile, we also found miR-212-5p treatment somehow alleviated apoptosis in PD model, which might have some underlying mechanisms. Moreover, miR-212-5P highly selectively inhibited SIRT2 expression over SIRT1. These findings give us an insight into the potential development of miR-212-5p-based SIRT2 inhibitor in therapeutic avenues for PD.

## Materials and Methods

The study protocol was approved by the Institutional Animal Care and Use Committee of Nanjing Medical University.

### Animal Model

Twelve-week-old male C57BL/6 mice were randomly divided into four groups: negative control with saline-treated group, negative control with MPTP-treated group, miR-212-5p with saline-treated group, and miR-212-5p with MPTP-treated group. All animals were kept in cages with constant temperature (25°C) and humidity and were exposed to a 12/12-h light–dark cycle with unrestricted access to tap water and food. Mice received 25 mg/kg MPTP (Sigma, St. Louis, MO, United States) subcutaneously once a day for 7 days. Saline control mice were treated with the same volume of saline. Animals were sacrificed at 5 days after the last injection of MPTP or saline.

### Treatment of PD Mice Model With miR-212-5p Mimics

MiR-212-5p mimics and negative control were treated 3 days before MPTP injection. Anesthetized mice were positioned in a stereotaxic apparatus, and 2.5 μl of phosphate-buffered saline containing 0.5 nmol of miR-212-5p mimics or negative control (GenePharm, Shanghai, China) was injected over 10 min by a 33G Hamilton syringe (0.25 μl/min) into the lateral ventricle at stereotactic coordinates (millimeters from bregma): anterior-posterior (AP) = −0.3 mm, mediolateral (ML) = −0.13 mm, and dorsoventral (DV) = −0.47 mm from the skull surface as reported (Block et al., 2007). To visualize the distribution of miR-212-5p, mice were injected with Cy3-labeled miR-212-5p mimics.

### Transmission Electron Microscopic Analysis

Mice were perfused with 2.5% glutaraldehyde and 2% paraformaldehyde. A small portion (∼1 mm^3^) of the hippocampus was sectioned and incubated for 2 h at 4°C in the same fixative. Specimens were postfixed in 1% osmium tetroxide, stained in aqueous uranyl acetate, and then dehydrated and embedded in epoxy resin. Ultrathin sections were stained using lead citrate and examined with transmission electron microscope (JEM-1010, Tokyo, Japan). All experiments and photographs of TEM were supported by the grant from the center of forecasting and analysis of Nanjing Medical University.

### Immunofluorescence, Unbiased Stereology, and TUNEL Staining

For frozen samples, mice were perfused transcardially with 4% paraformaldehyde. Brains were extracted, post-fixed, dehydrated, embedded in OCT (Tissue-Tek), and cryosectioned at 30 μm per slice. For immunofluorescence, slides were incubated with the indicated primary antibodies at 4°C overnight, then washed and incubated in secondary fluorescent antibodies, followed by mounting in Prolong Gold Antifade with DAPI (Life Technologies, Cat P36931) before imaging. For *in vivo* cell quantification studies, the number of TH^+^ neurons in the SNpc of the midbrain was assessed using the optical fractionator (Stereo Investigator 7, MBF Bioscience, Williston, VT, United States) as previously reported (Han et al., 2018). All stereological analyses were performed under the 200× objective of an Olympus BX52 microscope (Olympus America Inc., Melville, NY, United States). The brain slices and cells were sampled to measure TUNEL positive with a TUNEL BrightGreen Apoptosis Detection Kit (Vazyme) following the manufacturer’s instructions. Briefly, 30 μm frozen sections were incubated with proteinase K (20 mg/ml) for 10 min at room temperature. The sections were incubated in the TdT buffer including BrightGreen Labeling Mix and Recombinant TdT Enzyme for 1 h at 37°C. Images were captured by Nikon fluorescence microscope (TE2000-S).

### Cell Culture and Treatment

The SH-SY5Y neuroblastoma cell line was cultured in Dulbecco’s modified Eagle’s medium (DMEM) supplemented with 2 mM L-glutamine, penicillin (20 units/ml), streptomycin (20 mg/ml), and 10% (vol/vol) heat-inactivated fetal calf serum (GIBCO, Gaithersburg, MD, United States). Cells were maintained at 37°C in a saturated humidity atmosphere containing 95% air and 5% CO_2_. Cells were treated with MPP^+^ (Sigma, St. Louis, MO, United States) at the dosage of 100 μM for 24 h.

### Oligonucleotide and Plasmid Transfection

MiR-212-5p mimics (sense 5′-ACCUUGGCUCUAGACUGCUUACU-3′, antisense 5′-UAAGCAGUCUAGAGCCAAGGUUU-3′), control miR (sense 5′-UUCUCCGAACGUGUCACGUTT-3′, antisense 5′-ACGUGACACGUUCGGAGAATT-3′), anti-miR-212-5p (sense 5′-UUCUCCGAACGUGUCACGUTT-3′, antisense 5′-ACGUGACACGUUCGGAGAATT-3′), and anti-miR control (sense 5′-UUCUCCGAACGUGUCACGUTT-3′) were purchased from GenePharm (Shanghai, China). MiR-212-5p, anti-miR-212-5p and corresponding control miR were complexed, respectively, with Lipofectamine 3000 (Invitrogen Life Technologies, United States) according to manufacturer’s instructions. SH-SY5Y cells were transfected with miR-212-5p mimics, miR-212-5p inhibitor, and corresponding control miR for 6 h, followed by followed by stimulation with MPP^+^ (100 μM) for 24 h.

SH-SY5Y cells were transfected with pcDNA3.1(-)-3HA-p53 plasmid (0.5 μg, 1 μg, and 2 μg, respectively) using Lipofectamine 3000 described previously (Shan et al., 2016). The pcDNA3.1(-)-Flag is used as the mock. In addition, SH-SY5Y cells were transfected with 0.5 μg mTag-Wasabi-LC3 plasmid and manipulated in accordance with the instruction of Lipofectamine 3000. Six hours after transfection, transfection reagents were replaced by nutrient medium containing 10% FBS and cultured overnight.

### Immunocytochemistry

Cells plated on coverslips were fixed in 4% paraformaldehyde in PBS for 10 min. Cells were then blocked with 5% BSA containing 0.1% Triton X for 1 h. After that, the cells were incubated with primary antibodies (anti-SIRT2, Abcam; anti-LC3, Cell Signaling Technology; anti-p53, Invitrogen) overnight at 4°C. Afterward, cells were incubated with the Alexa 488 and 555 coupled secondary antibodies (Invitrogen) for 1 h at room temperature followed by counterstained with DAPI. Images were captured by Nikon fluorescence microscope (TE2000-S).

### Western Blotting

Tissues and cell protein lysates were fractionated by a nucleoprotein extraction kit (KeyGen BioTECH, Nanjing, China), and protein concentrations were quantified by BCA method (Biyuntian kit, Beyotime, Shanghai, China). Equivalent amounts of extracted proteins (30 μg) were resolved on SDS-PAGE and electroblotted to PVDF membrane (Amersham Biosciences). After blocking the background staining with 5% BSA in TBST, the membranes were incubated in primary antibodies against SIRT1 (1:1000, Abcam), SIRT2 (1:1000, Abcam), LC3B (1:1000, Cell Signaling Technology), p62 (1:1000, Cell Signaling Technology), p53 (1:1000, Invitrogen), ace-p53 Lys379 (1:500, Cell Signaling Technology), BAX (1:1000, Invitrogen), Bcl-2 (1:1000, Invitrogen), caspase-3 (1:500, Cell Signaling Technology), DAT (1:1000, Santa Cruz), β-actin (1:2000, Sigma), and anti-HA (1:1000, Invitrogen) overnight at 4°C. Immunoreactive proteins were detected using HRP conjugated secondary antibodies and an ECL kit (Amersham Biosciences) according to the manufacturer’s instructions. The membranes were scanned and analyzed in an Image Quant LAS 4000 Chemiluminescence Imaging System (GE Healthcare, United States). Optical densities of bands were analyzed by using ImageJ. Protein levels, quantified by the ratio between each immunoreactive band and the levels of β-actin (for cytoplasmic protein) or H3 (for nucleoprotein), were expressed as a percentage of vehicle-treated control.

### qRT-PCR Analysis of mRNA and MicroRNA Expression

Total RNA was prepared using TRIZOL reagent (Invitrogen Life Technologies, United States). Reverse transcription of total RNA using TaKaRa Master Mix (TaKaRa, Japan). The primers were purchased from GENEray (Shanghai, China). Real-time qPCR was carried out using SYBR Green Master Mix (Applied Biosystems) in a StepOnePlus instrument (Applied Biosystems). The primers used for qPCR were as follows: SIRT2 (sense 5′-CCTCCTTGCAGGGACGTGG-3′, antisense 5′-GCTGTCACTGGGGTTTCTCC-3′); GAPDH (sense 5′-AATGGGCAGCCGTTAGGAAA-3′, antisense 5′-GCGCCCAATACGACCAAATC-3′). MicroRNA specific primers were purchased from GeneCopoeia. The relative expression was calculated using the ΔCT method as described elsewhere and normalized to uniformly expressed U6. All qRT-PCRs were performed in triplicates, and the data are presented as mean ± standard error (SEM).

### Dual Luciferase Reporter Assays

Luciferase reporter assays were performed using the psiCHECK2-3′UTR vector described previously (Fan et al., 2016). Briefly, cells were grown to 50% confluence in 24-well plates and co-transfected with psiCHECK2-SIRT2-3′UTR or Mut-SIRT2-3′UTR (GENEray Biotechnology, Shanghai, China) plus miR-212-5p mimics or negative control mimics. Cells were incubated with a transfection complex for 24 h followed by luciferase reporter assay using the Dual Luciferase Assay System (Promega, Madison, WI, United States). Renilla luciferase activity was normalized to firefly luciferase activity. Cell lysates were subjected to luciferase activity measurement according to the manufacturer’s instructions.

### Cell Viability Assay

Cells were cultured in the 96-well plate and two extra wells were set for each group. After treatment, we discarded the medium and performed according to the instruction of EnoGeneCell^TM^ Counting Kit-8 (CCK-8). 10 μL CCK-8 solution was added in one well and the plate was put in the incubator for about 3 h. We measured the wavelength of each well at 450 nm by microplate reader when time was over.

### Lactate Dehydrogenase (LDH) Assay

Cells were planted in 96-well plate at 5,000 cells/well, and treated as described above. Culture medium was collected to measure LDH levels with an assay kit (Nanjing Jiancheng Bioengineering Institute) following the manufacturer’s instructions. The samples were quantified at 440 nm with a spectrophotometric plate reader.

### Hoechst Staining

To quantify apoptotic SH-SY5Y cells, monolayer cells were fixed and stained with Hoechst 33342 (Sigma, St Louis, MO, United States). The morphological features of apoptosis (cell shrinkage, chromatin condensation, and fragmentation) were monitored by fluorescence microscopy (Olympus BX 60, Tokyo, Japan). At least 300 cells from 12 randomly selected fields per dish were counted, and each treatment was performed in triplicate.

### SIRT2 Activity Assay

The SIRT2 activity was determined by the instrument of SIRT2 direct fluorescent screening assay kit (Cayman, United States). Generally speaking, Cayman’s SIRT2 direct fluorescent screening assay provides a fluorescence-based method for screening SIRT2 inhibitor or activators. The procedure requires only two steps. In the first step, the substrate is incubated with human recombinant SIRT2 along with its co-substrate NAD^+^. Deacetylation sensitizes the substrate such that treatment with the developer in the second step release a fluorescent product. The fluorophore can be analyzed with an excitation wavelength of 350 nm and an emission wavelength of 465 nm. Subtract each inhibitor sample value from the 100% initial activity sample value. Divide the result by the 100% initial activity value and then multiply by 100 to give the percent of inhibition.

### Statistics

The data are presented as mean ± SEM. Statistical differences were evaluated using Student’s *t*-test for comparisons of two groups or analysis of variance (ANOVA) and appropriate *post hoc* analyses for comparisons of more than two groups. *P* < 0.05 was considered significant.

## Results

### SIRT2 Protein Expression Is Increased in PD Experimental Model

**FIGURE 1:**
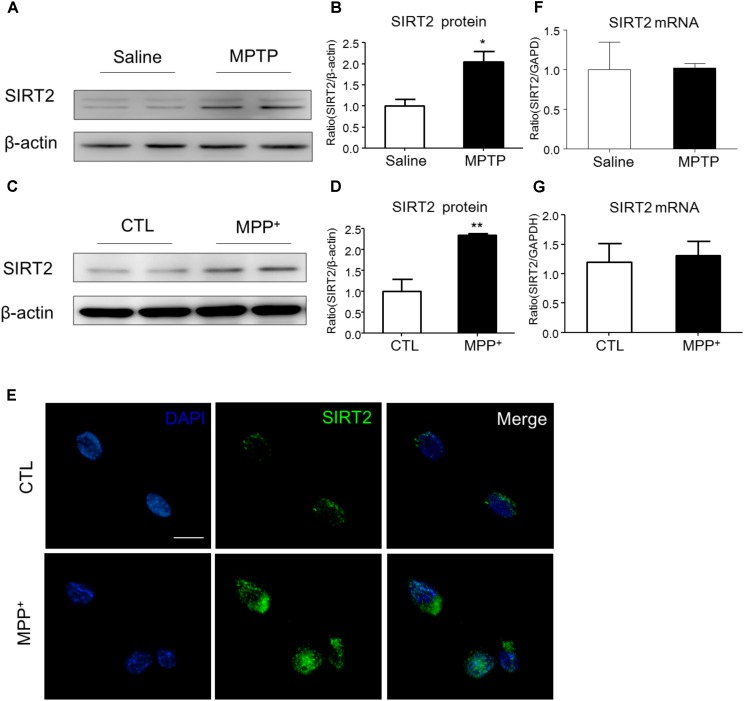
The expression patterns of SIRT2 in PD model. **(A–D)** Western blotting analysis showed that SIRT2 protein level increased by MPTP treatment or MPP^+^ stimulating, respectively. **(E)** Immunohistochemistry staining showed a significant increment of SIRT2 in SH-SY5Y cells by MPP^+^ stimulation, compared with controls. *Scale bar*: 40 μm. **(F,G)** Quantitative RT-PCR analysis for SIRT2 mRNA level showed no significant difference in midbrain tissues and SH-SY5Y cells under treatment. Data are presented as the mean ± SEM for each group of five mice or three independent *in vitro* experiments. ^∗^*p* < 0.05, ^∗∗^*p* < 0.01 versus corresponding control group.

To examine the expression of SIRT2 in PD model, we detected SIRT2 protein and mRNA levels both *in vivo* and *in vitro*. Compared to the saline-treated mice, MPTP injection highly increased the expression of SIRT2 in the midbrain (**Figures 1A,B**). Similarly, SIRT2 protein level was markedly up-regulated with MPP^+^ stimulation in SH-SY5Y cells (**Figures 1C,D**). Consistent with the result of Western blotting, immunocytochemistry staining showed SIRT2 was highly expressed in SH-SY5Y cells by MPP^+^ stimulation (**Figure 1E**). However, there was no significant difference in SIRT2 mRNA level both *in vivo* and *in vitro* (**Figures 1F,G**), which indicated there might have post-transcriptional regulation of SIRT2 expression.

### SIRT2 Is a Target Gene of miR-212-5p

**FIGURE 2:**
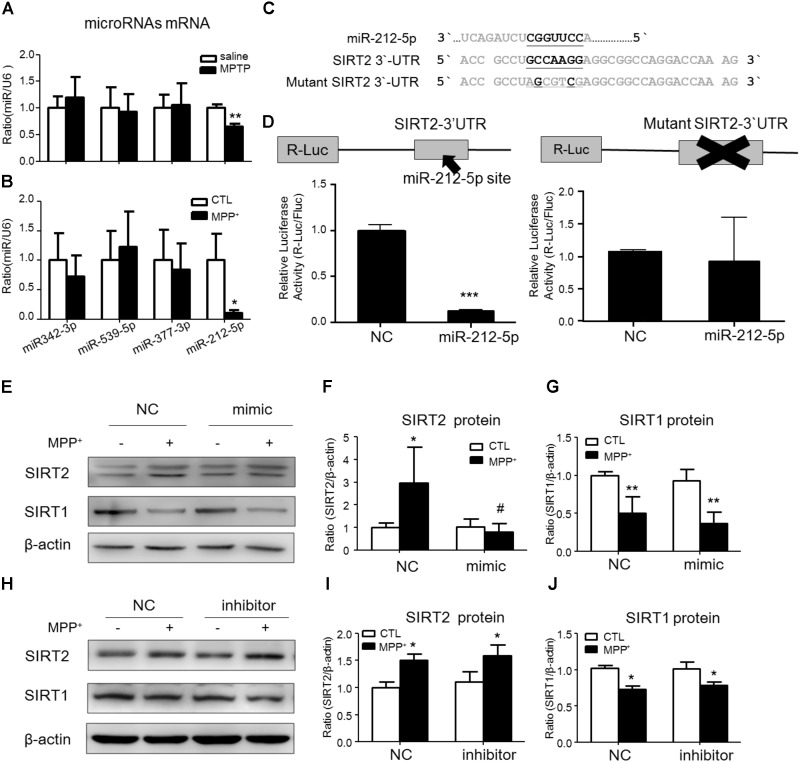
SIRT2 is a target gene of miR-212-5p. **(A,B)** Quantitative RT-PCR analysis detected some predicted SIRT2-associated microRNAs expression. Results showed miR-212-5p significantly decreased in PD model both *in vivo* and *in vitro*. **(C)** The predicted binding sites of miR-212-5p in the 3′UTR of SIRT2, and schematic description of the base-pairing interaction between miR-212-5p and SIRT2 mRNA. **(D)** Luciferase reporter assays confirm that SIRT2 is a direct target gene of miR-212-5p in HEK293T cells. **(E–G)** Transfection of miR-212-5p into SH-SY5Y cells significantly reduced SIRT2 expression but failed to affect SIRT1 expression. **(H–J)** Anti-miR-212-5p up-regulated SIRT2 expression without significant difference, but had no effect on SIRT1 expression. Data are presented as the mean ± SEM from three independent experiments. ^∗^*p* < 0.05, ^∗∗^*p* < 0.01, ^∗∗∗^*p* < 0.001 versus control or NC-control group; ^#^*p* < 0.05 versus NC plus MPP^+^ group. NC, negative control.

To study the post-transcriptional regulation of SIRT2, we predicted some miRNAs directly binds in 3′-untranslated region (3′-UTR) of SIRT2 mRNA by targetscan website^1^. Among these miRNAs, we found that miR-212-5p was obviously decreased in the midbrain of MPTP-induced PD mice and MPP^+^-treated SH-SY5Y cells (**Figures 2A,B**). Thus, miR-212-5p was selected for further experimental verification. As predicted, miR-212-5p directly binds in 3′UTR of SIRT2 mRNA (**Figure 2C**). To further verify our prediction, we inserted the 3′-UTR sequence of SIRT2 mRNA containing miR-212-5p seed sequence into a dual luciferase reporter construct. We discovered that miR-212-5p could suppress the expression of Renilla luciferase (R-Luc) through the SIRT2 3′-UTR. In contrast, the mutated miR-212-5p seed sequence within the R-Luc-SIRT2-3′-UTR reporter abolished the miR-212-5p-mediated suppression of R-Luc reporter activity (**Figure 2D**). Next, we detected the effects of altered miR-212-5p on endogenous SIRT2 and SIRT1 expression in SH-SY5Y cells. The results showed that the transfection of miR-212-5p significantly reduced SIRT2 protein level (two-way ANOVA, MPP^+^: *F*_1,20_ = 5.901, *p* = 0.0247; genotype: *F*_1,20_ = 6.868, *p* = 0.0164; interaction: *F*_1,20_ = 7.200, *p* = 0.0143) (**Figures 2E,F**), whereas anti-miR-212-5p transfection failed to up-regulate SIRT2 expression (two-way ANOVA, MPP^+^: *F*_1,20_ = 9.710, *p* = 0.0143; genotype: *F*_1,20_ = 0.3253, *p* = 0.5841; interaction: *F*_1,20_ = 0.002814, *p* = 0.9590) (**Figures 2H,I**). One thing is noted that neither miR-212-5p nor anti-miR-212-5p transfection altered SIRT1 expression (two-way ANOVA, MPP^+^: *F*_1,20_ = 11.80, *p* = 0.0089; genotype: *F*_1,__20_ = 0.4679, *p* = 0.5133; interaction: *F*_1,20_ = 7.200, *p* = 0.8399) (**Figure 2G**) (two-way ANOVA, MPP^+^: *F*_1,20_ = 17.25, *p* = 0.0006; genotype: *F*_1,20_ = 0.3969, *p* = 0.5366; interaction: *F*_1,20_ = 0.3969, *p* = 0.4583) (**Figure 2J**). These results indicate that miR-212-5p negatively regulates SIRT2 expression, but no SIRT1.

### MiR-212-5p Promotes MPP^+^-Induced Autophagy in Cultured SH-SY5Y Cells

**FIGURE 3:**
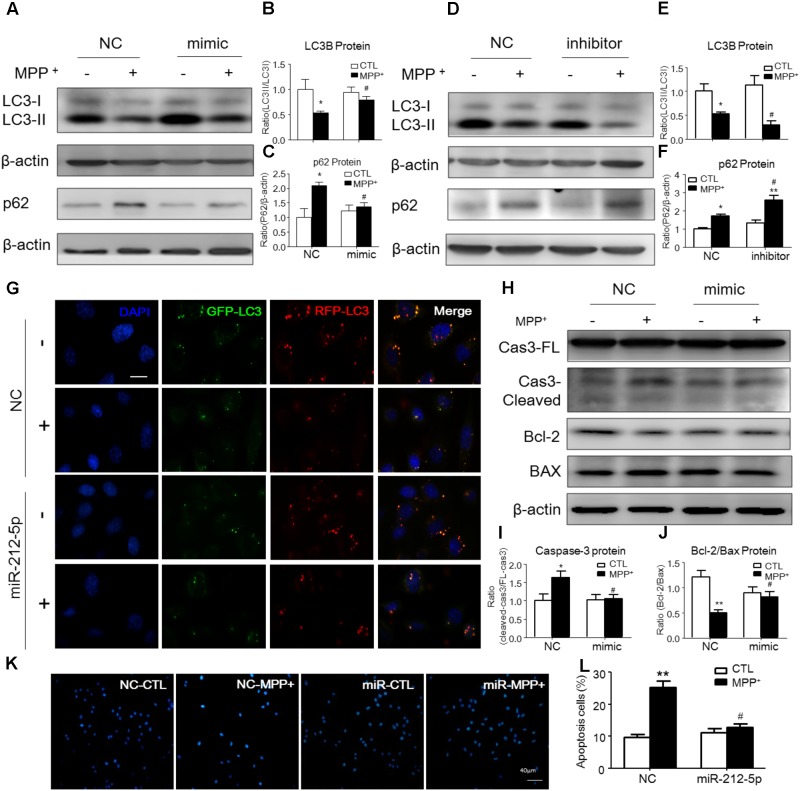
MiR-212-5p promotes autophagy and inhibits apoptosis in SH-SY5Y cells. **(A–C)** Western blotting and quantitative analysis of LC3B and P62 revealed that transfection of miR-212-5p into SH-SY5Y cells activated autophagy. **(D–F)** In contrast, miR-212-5p inhibitor indeed blocked autophagy, which showed by Western blotting and quantitative analysis of autophagy markers. **(G)** Double immunofluorescence of GFP-LC3B (Green) and RFP-LC3B (Red). MPP^+^ blocked the autophagy flux in SH-SY5Y cells, which was reversed by miR-212-5p transfection. *Scar bar*: 40 μm. **(H–J)** Transfection of miR-212-5p into SH-SY5Y cells reduced apoptosis, Western blotting and quantitative analysis of cleaved caspase-3, Bcl2 and BAX. **(K,L)** Apoptotic cells were stained by Hoechst 33342 and analysis of positive cells. *Scale bar*: 80 μm. Data is presented as the mean ± SEM from three independent experiments. ^∗^*p* < 0.05, ^∗∗^*p* < 0.01, ^∗∗∗^*p* < 0.001 versus control or NC-control group; ^#^*p* < 0.05, ^##^*p* < 0.01, ^###^*p* < 0.01 versus NC plus MPP^+^ group.

To explore whether miR-212-5p has a functional role on MPP^+^-induced cytotoxicity, we detected the effect of miR-212-5p on autophagy. As determined by Western blotting, we found that LC3-II level in the MPP^+^-treated SH-SY5Y cells was significantly decreased compared with the PBS-treated cells. Meanwhile, the p62, an important autophagy substrate, was highly elevated. In contrast, miR-212-5p transfection could increase LC3-II (two-way ANOVA, MPP^+^: *F*_1,20_ = 43.43, *p* = 0.0001; genotype: *F*_1,20_ = 4.653, *p* = 0.0434; interaction: *F*_1,20_ = 7.561, *p* = 0.0123) (**Figures 3A,B**) and decrease p62 expression (two-way ANOVA, MPP^+^: *F*_1,18_ = 16.82, *p* = 0.0173; genotype: *F*_1,18_ = 4.393, *p* = 0.0514; interaction: *F*_1,18_ = 9.321, *p* = 0.0072) (**Figures 3A,C**). On the other hand, miR-212-5p inhibitor aggravated the impaired autophagy, as shown by decreased LC3B-II (two-way ANOVA, MPP^+^: *F*_1,18_ = 8.23, *p* = 0.0121; genotype: *F*_1,18_ = 5.626, *p* = 0.0278; interaction: *F*_1,18_ = 1.256, *p* = 0.2757) (**Figures 3D,E**) and increased p62 expression (two-way ANOVA, MPP^+^: *F*_1,18_ = 27.53, *p* = 0.0002; genotype: *F*_1,18_ = 13.97, *p* = 0.0028; interaction: *F*_1,18_ = 4.316, *p* = 0.0519) (**Figures 3D,F**). These results indicate that autophagy is inhibited by MPP^+^-induced cytotoxicity, which is promoted by miR-212-5p transfection in SH-SY5Y cells. To explore whether the accumulation of autophagosomes is due to decreased fusion of autophagosomes with lysosomes or increased autophagosomal formation, we used mTagRFP-Wasabi-LC3 plasmid to detect the autophagic flux. Once the maturation of an autolysosome occurs, only the RFP signal can be exhibited because it is resistant to the lysosomal acidic/proteolytic environment. The immunofluorescence images showed that MPP^+^ blocked the autophagic flux, but activated by miR-212-5p transfection (**Figure 3G**).

### MiR-212-5p Regulates p53 Sub-Localization via SIRT2 Activity in SH-SY5Y Cells

**FIGURE 4:**
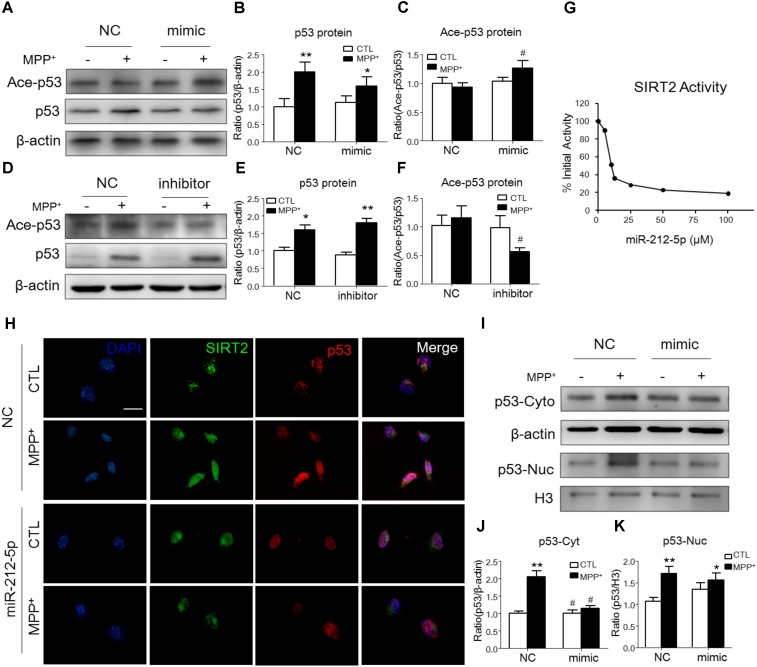
MiR-212-5p regulates p53 sub-localization via SIRT2 activity in PD model. **(A,B)** Western blotting and quantitative analysis showed that the total p53 expression was highly increased by MPP^+^ stimulating, which was somehow inhibited by miR-212-5p mimic treatment. **(D,E)** In contrast, miR-212-5p inhibitor indeed enhanced the increased total p53 expression. Since p53 is a key deacetylation substrate of SIRT2, we detected the acetylation of p53 by Western blotting. Results showed that miR-212-5p regulated the level of acetylated p53 **(C,F)**. **(G)** SIRT2 activity assay revealed that miR-2125p could suppress the deacetylation potential of SIRT2. **(H)** Double immunofluorescence of SIRT2 (green) and p53 (red). *Scale bar*: 40 μm. Consistent with previous results, SIRT2 and p53 were highly increased under MPP^+^ treatment, which was inhibited by miR-212-5p transfection. **(I–K)** Western blotting and quantitative analysis showed that p53 was up-regulated both in cytoplasm and nucleus. miR-212-5p transfection suppressed the expression of p53, especially in cytoplasm. ^∗^*p* < 0.05, ^∗∗^*p* < 0.01 versus control or NC-control group; ^#^*p* < 0.05, ^##^*p* < 0.01 versus NC plus MPP^+^ group.

It is well known, SIRT2 plays biological function as a histone deacetylate. P53 is one of the important deacetylation substrates. Thus, we observed the acetylation level of p53 and SIRT2 activity to evaluate the function of SIRT2. As shown by Western blotting, the p53 expression was highly increased, and miR-212-5p transfection could alter the expression of p53 (two-way ANOVA, MPP^+^: *F*_1,20_ = 12.09, *p* = 0.0023; genotype: *F*_1,20_ = 2.96, *p* = 0.0277; interaction: *F*_1,20_ = 4.709, *p* = 0.0416) (**Figures 4A,B,D,E**). However, there was no significant difference of p53 acetylation level in MPP^+^-treatment cells. Interestingly, miR-212-5p up-regulated the acetylation level of p53 (two-way ANOVA, MPP^+^: *F*_1,20_ = 0.6008, *p* = 0.5580; genotype: *F*_1,20_ = 6.253, *p* = 0.0212; interaction: *F*_1,20_ = 3.136, *p* = 0.0654) (**Figures 4A,C**) and anti-miR-212-5p down-regulated the acetylation level of p53 (two-way ANOVA, MPP^+^: *F*_1,12_ = 2.545, *p* = 0.1347; genotype: *F*_1,12_ = 5.295, *p* = 0.0386; interaction: *F*_1,12_ = 2.509, *p* = 0.1372) (**Figures 4D,F**). Considering SIRT2 as a histone deacetylases, we also detected the activity which was regulated by miR-212-5p transfection. As shown in **Figure 4G**, at the dosage of 25 μM, miR-212-5p could decrease almost 71.54% activity of SIRT2. To reveal the expression and localization of SIRT2 and p53 under MPP^+^-treated SH-SY5Y cells, we observed them by immunochemistry double staining. As shown in the images, MPP^+^ both increased SIRT2 and p53 expression in the whole cells. MiR-212-5p obviously decreased SIRT2 expression and cytosolic p53 expression (**Figure 4H**). Western blot results showed that p53 expression both in cytoplasm and nuclear was up-regulated in SH-SY5Y cells under MPP^+^ stimulation, and miR-212-5p significantly reversed the cytosolic up-regulation of p53 (two-way ANOVA, MPP^+^: *F*_1,20_ = 28.74, *p* = 0.0001; genotype: *F*_1,20_ = 16.83, *p* = 0.0012; interaction: *F*_1,20_ = 17.03, *p* = 0.0012) (**Figures 4I,J**), but no significant effect on the nuclear p53 expression (two-way ANOVA, MPP^+^: *F*_1,20_ = 12.53, *p* = 0.0022; genotype: *F*_1,20_ = 1.278, *p* = 0.2723; interaction: *F*_1,20_ = 4.711, *p* = 0.0428) (**Figures 4I,K**).

### P53 Transfection Abolished the SIRT2-Related Programmed Cell Death in SH-SY5Y Cells

**FIGURE 5:**
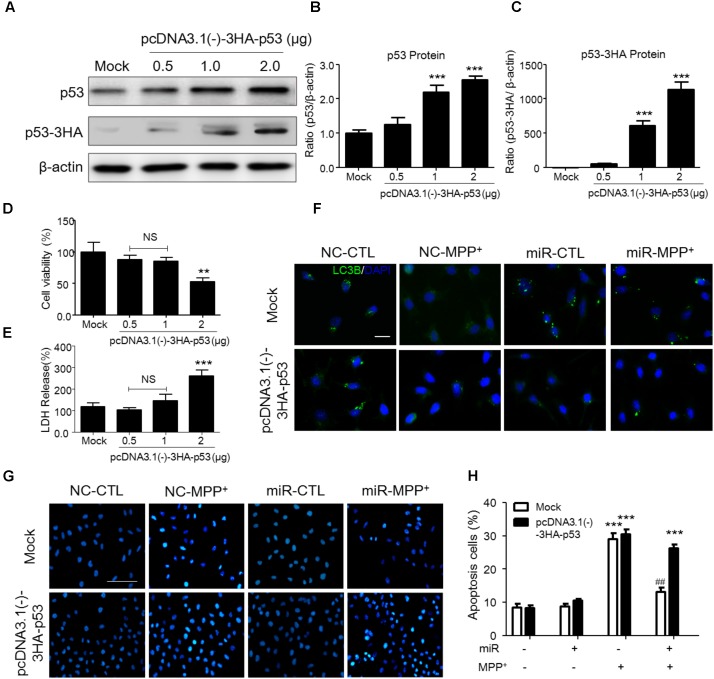
P53 transfection abolished the SIRT2-related programmed cell death in SH-SY5Y cells. **(A–C)** According to p53 as a key transfactor dealing with the programmed cell death, to detect the realistic role of p53 regulated by SIRT2 in this study, we transfected pcDNA3.0-Flag-p53 plasmid to SH-SY5Y cells. Western blotting and quantitative analysis showed that 1 μg and 2 μg plasmid transfection highly increased the p53 expression. **(D,E)** CCK-8 and LDH release assay showed that 0.5 μg and 1 μg plasmid transfection had no effects on cell viability. Thus, we transfected 1 μg pcDNA3.0-Flag-p53 plasmid to SH-SY5Y cells in the further study. **(F)** Immunohistochemistry staining showed a significant increment of LC3 (green) under MPP^+^ stimulation could suppressed by miR-212-5p transfection, which was blocked by p53 overexpression. *Scar bar*: 40 μm. **(G,H)** Apoptotic cells were stained by Hoechst 33342 and analysis of positive cells. MiR-212-5p decreased the apoptotic cells by MPP^+^ stimulation, which was blocked by p53 overexpression. *Scale bar*: 80 μm. ^∗^*p* < 0.05, ^∗∗^*p* < 0.01, ^∗∗∗^*p* < 0.01 versus Mock or Mock with NC control group; ^##^*p* < 0.01 versus pcDNA3.0-Flag-p53 plasmid plus MPP^+^ group.

To demonstrate whether the function of miR-212-5p is dependent on p53, we transfected pcDNA3.0-Flag-p53 into SH-SH5Y cells. We adopted three different plasmid concentrations (0.5, 1, and 2 μg) and found that 1 μg plasmid was efficient to increase p53 expression (**Figures 5A–C**) without impact on the survival of SH-SY5Y cells (**Figures 5D,E**). Therefore, 1 μg plasmid was used for following experiments. Compared to the MPP^+^ -treated group, miR-212-5p activated LC3-II positive puncta, which was inhibited by pcDNA3.0-Flag-p53 transfection (**Figure 5F**). In addition, MPP^+^ induced apoptosis, and miR-22-5p could alleviate the apoptosis induced by MPP^+^ (**Figures 5G,H**). In contrast, pcDNA3.0-Flag-p53 transfection abolished anti-apoptotic effect of miR-22-5p (**Figures 5G,H**). Taken together, p53 is required for miR-212-5p against MPP^+^-induced PCD.

### MiR-212-5p Protects DA Neuron Against Degeneration in MPTP PD Model Mice by Promoting p53-Dependent Autophagy

**FIGURE 6:**
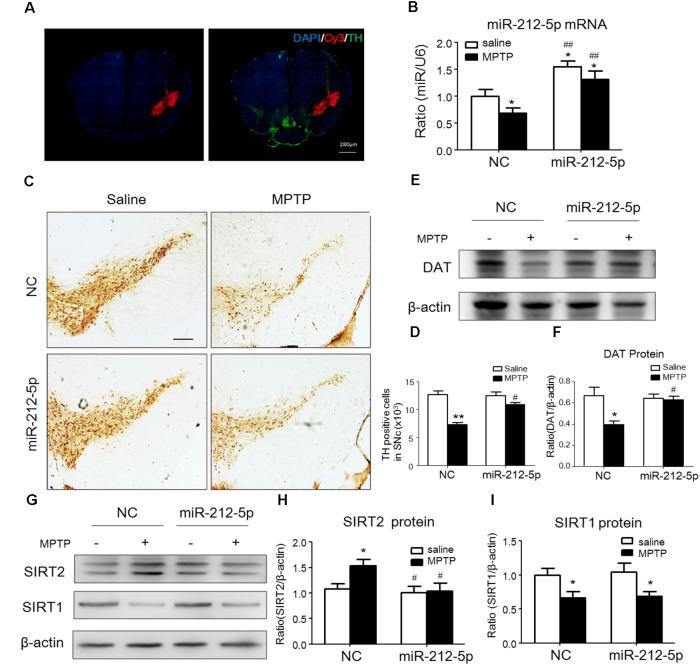
MiR-212-5p decreases SIRT2 expression and attenuates DA neuron loss *in vivo*. **(A)** Fluorescent image of Cy3-labeled miR-212-5p (red) and TH-marked neurons (green) was observed in the SNc of mice. **(B)** RT-PCR analysis of miR-212-5p. **(C,E)** Representative microphotographs and quantitative analysis of TH in the SNc of MPTP injured mice. *Scar bar*: 200 μm. **(D,F)** Western blotting and quantitative analysis showed that DAT was decreased by MPTP-induced PD mice model, which was reversed by miR-212-5p treatment. **(G,H)** Western blotting and quantitative analysis of SIRT2 and SIRT1. Consistent with the previous founds, SIRT2 was increased and SIRT1 was decreased in MPTP-induce PD mice model. MiR-212-5p treatment decreased SIRT2 expression with no influence on SIRT1. Data are presented as the mean ± SEM for each group of five mice, Two-way ANOVA, ^∗^*p* < 0.05, ^∗∗^*p* < 0.01 versus NC with saline treatment; ^#^*p* < 0.05, ^##^*p* < 0.01, ^###^*p* < 0.001 versus NC with MPTP-treatment.

**FIGURE 7:**
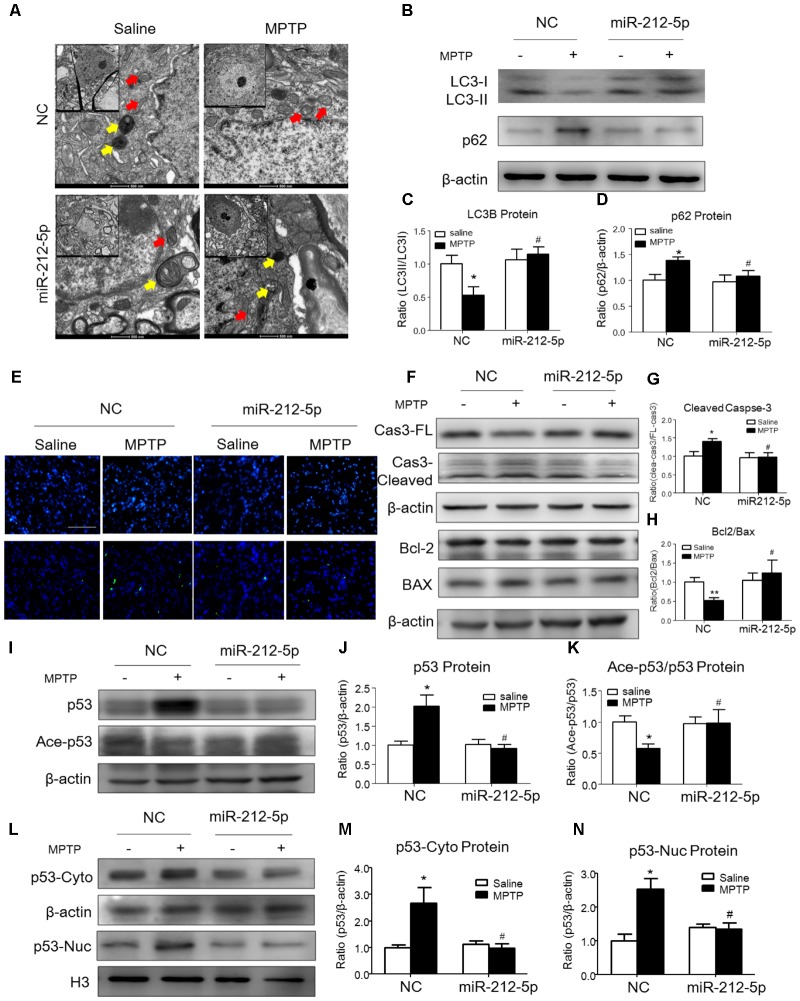
MiR-212-5p promotes p53-dependent autophagy and inhibits apoptosis *in vivo*. **(A)** Transmission electron microscope of midbrain in mice. Yellow arrow means autolysosome, red arrow means mitochondrion. **(B–D)** Representative immunoblots and quantification of midbrain extracts for analysis of LC3B and P62 in mice of both normal and MPTP-treatment. **(E)** TUNEL staining of midbrain in mice. **(F–H)** Representative immunoblots and quantification of midbrain extracts for analysis of cleaved caspase-3, Bcl-2 and BAX. **(I–K)** Western blotting and quantitative analysis showed that miR-212-5p decreased total p53 and increased ace-p53 expression in the midbrain of mice. **(L–N)** Western blotting and quantitative analysis revealed that miR-212-5p decreased both cytosolic and nuclear p53 expression. Data are presented as the mean ± SEM for each group of five mice. ^∗^*p* < 0.05, ^∗∗^*p* < 0.01 versus NC with saline treatment; ^#^*p* < 0.05, ^##^*p* < 0.01 versus NC with MPTP-treatment.

We further investigated the role of miR-212-5p in the pathogenesis of MPTP-induced PD mice model. Firstly, we confirm miR-212-5p expression by exogenous injection. As shown in immunofluorescence, the exogenous miR-212-5p was highly expressed in SNc labeled by Cy3, which was co-located with TH-marked neurons in SNc (**Figure 6A**). MiR-212-5p increased its expression in the midbrain of MPTP-treated mice by real-time PCR (two-way ANOVA, MPTP: *F*_1,20_ = 4.873, *p* = 0.0398; genotype: *F*_1,20_ = 22.37, *p* = 0.0001; interaction: *F*_1,20_ = 0.09531, *p* = 0.7609) (**Figure 6B**). Then we evaluated the protective effects of miR-212-5P on DA neurons. Obviously, injection of miR-212-5p dramatically rescued the loss of TH^+^ neuron in the SNc of MPTP-treated mice (two-way ANOVA, MPTP: *F*_1,20_ = 43.44, *p* < 0.0001; genotype: *F*_1,20_ = 10.12, *p* = 0.0047; interaction: *F*_1,20_ = 13.03, *p* = 0.0017) (**Figures 6C,D**). In addition, miR-212-5p also elevated the DAT expression under MPTP treatment (two-way ANOVA, MPTP: *F*_1,20_ = 9.166, *p* = 0.0067; genotype: *F*_1,20_ = 4.704, *p* = 0.0423; interaction: *F*_1,20_ = 7.546, *p* = 0.0124) (**Figures 6E,F**). These findings suggest that miR-212-5p may exert a crucial role in the pathogenesis of PD. To confirm whether miR-212-5p is a specific inhibitor of SIRT2, we detected SIRT2 expression by Western blotting. Compared to the MPTP-treated NC mice, miR-212-5p rescued the elevated SIRT2 expression (two-way ANOVA, MPTP: *F*_1,20_ = 4.806, *p* = 0.0410; genotype: *F*_1,20_ = 6.470, *p* = 0.0198; interaction: *F*_1,20_ = 3.841, *p* = 0.0649) (**Figures 6G,H**) without influence on SIRT1 expression (two-way ANOVA, MPTP: *F*_1,20_ = 6.964, *p* = 0.0179; genotype: *F*_1,20_ = 0.01939, *p* = 0.8910; interaction: *F*_1,20_ = 0.3230, *p* = 0.5777) (**Figures 6G,I**), which is consistent with the *in vitro* study. To verify that miR-212-5p is responsible for autophagy by targeting SIRT2 in PD mice, we next detected by TEM and Western blotting. Under TEM, we observed several autophagosomes in cytoplasm which located close to neuron in the mice of miR-212-5p treatment. We also found mitochondria were impaired in MPTP-treated mice while the impairment was alleviated after miR-212-5p treatment (**Figure 7A**). Consistent with the previous study *in vitro*, the classic autophagic markers LC3-II (two-way ANOVA, MPTP: *F*_1,20_ = 4.525, *p* = 0.0444; genotype: *F*_1,20_ = 4.551, *p* = 0.0438; interaction: *F*_1,20_ = 7.781, *p* = 0.0104) (**Figures 7B,C**) and p62 (two-way ANOVA, MPTP: *F*_1,20_ = 7.996, *p* = 0.0144; genotype: *F*_1,20_ = 6.945, *p* = 0.0159; interaction: *F*_1,20_ = 4.521, *p* = 0.0461) (**Figures 7B,D**) were dysregulated in MPTP-treated mice, which regulated by miR-212-5p treatment. Mechanismly, we detected the acetylation of p53 because p53 as an important deacetylation substrate of SIRT2. As shown by Western blotting, total p53 protein level was highly increased in MPTP PD mice, and miR-212-5p treatment suppressed p53 expression (two-way ANOVA, MPTP: *F*_1,18_ = 5.690, *p* = 0.0283; genotype: *F*_1,18_ = 8.025, *p* = 0.0110; interaction: *F*_1,18_ = 8.648, *p* = 0.0087) (**Figures 7I,J**). Meanwhile, miR-212-5p treatment increased the acetylation of p53 partly (two-way ANOVA, MPTP: *F*_1,18_ = 5.512, *p* = 0.0293; genotype: *F*_1,18_ = 4.524, *p* = 0.0461; interaction: *F*_1,18_ = 11.35, *p* = 0.0030) (**Figure 7K**). Considering p53 is a key duplex regulator in autophagy, we further found miR-212-5p regulated the p53 sub-localization *in vivo*. In detail, miR-212-5p decreased cytoplasmic p53 (two-way ANOVA, MPTP: *F*_1,18_ = 5.546, *p* = 0.0364; genotype: *F*_1,18_ = 6.631, *p* = 0.0243; interaction: *F*_1,18_ = 8.773, *p* = 0.0119) (**Figures 7L,M**) and increased nuclear p53 (two-way ANOVA, MPTP: *F*_1,18_ = 13.39, *p* = 0.0029; genotype: *F*_1,18_ = 4.789, *p* = 0.0475; interaction: *F*_1,18_ = 17.62, *p* = 0.0010) (**Figures 7L,N**) in the midbrain of MPTP-induced PD mice model. These data further demonstrate that miR-212-5p protects DA neurons against PD-like degeneration via activating p53-dependent autophagy by specifically inhibiting SIRT2.

### MiR-212-5p Ameliorates Apoptosis in PD Experimental Model

Since p53 is a critical transcription factor in PCD. We also detected whether miR-212-5p affected apoptosis. As shown by the Western blotting, the expression of cleaved caspase-3 was increased by MPP^+^ treatment in SH-SY5Y cells, whereas miR-212-5p obviously decreased MPP^+^-induced up-regulation of cleaved caspase-3. There was no difference in the expression of procaspase-3 either in MPP^+^ treatment alone or combined with miR-212-5p transfection (two-way ANOVA, MPP^+^: *F*_1,18_ = 6.643, *p* = 0.0185; genotype: *F*_1,18_ = 4.382, *p* = 0.0500; interaction: *F*_1,18_ = 6.768, *p* = 0.0175) (**Figures 3H,I**). MPP^+^ reduced the Bcl-2 and raised the Bax level in SH-SY5Y cells, which was remarkably alleviated by miR-212-5p treatment (two-way ANOVA, MPP^+^: *F*_1,18_ = 13.67, *p* = 0.0020; genotype: *F*_1,18_ = 9.547, *p* = 0.0333; interaction: *F*_1,18_ = 8.597, *p* = 0.0098) (**Figures 3H,J**). In addition, SH-SY5Y cells were stained with Hoechst 33342 to evaluate DNA fragmentation for determination of cell apoptosis. As shown in **Figures 3K,L** (two-way ANOVA, MPP^+^: *F*_1,48_ = 31.29, *p* < 0.0001; genotype: *F*_1,48_ = 12.70, *p* = 0.0008; interaction: *F*_1,48_ = 20.34, *p* < 0.0001), SH-SY5Y cells presented apoptosis features under MPP^+^ treatment, and miR-212-5p could decrease MPP^+^-induced apoptotic body formation. Taken together, these results suggest that miR-212-5p could alleviate MPP^+^-induced apoptosis of dopaminergic cells. Consistent with the results *in vitro*, the expression of cleaved caspase-3 (two-way ANOVA, MPTP: *F*_1,20_ = 4.712, *p* = 0.0422; genotype: *F*_1,20_ = 4.617, *p* = 0.0441; interaction: *F*_1,20_ = 5.561, *p* = 0.0287) (**Figures 7F,G**) was increased, and the ratio of Bcl-2/BAX was decreased in the MPTP-induced PD mice model, which was alleviated by miR-212-5p injection (two-way ANOVA, MPTP: *F*_1,20_ = 9.416, *p* = 0.0061; genotype: *F*_1,20_ = 4.273, *p* = 0.0419; interaction: *F*_1,20_ = 3.819, *p* = 0.0648) (**Figures 7F,H**). The TUNEL staining also confirmed the phenomenon. MiR-212-5P markedly inhibited apoptosis of neurons in SNc (**Figure 7E**). These data further suggests that miR-212-5p is closely related to accelerating neuronal apoptosis in the pathological process of PD.

## Discussion

Parkinson’s disease is an age-associated neurodegenerative disorder primarily known as a motor disorder due to the loss of dopaminergic neurons from the substantia nigra in the brain. Although there is still no cure or treatment available for PD, what is certain is that aging is the major risk factor. Therefore, any regimen that delays or interferes with the age-related decline in brain function would delay or prevent neurodegenerative diseases. SIRTs, a family of class III histone deacetylases, have beneficiary effects against age-related diseases by modulating a myriad of cellular processes, including energy metabolism, stress response, cell/tissue survival and malignancy (Donmez, 2012). In mammals, there are seven SIRTs (SIRT1–7), which are in different subcellular locations, and enzymatic activities, including the nucleus (SIRT1, SIRT6, and SIRT7), cytosol (SIRT2), and mitochondria (SIRT3, SIRT4, and SIRT5). Among them, SIRT1, SIRT2, and SIRT3 are robust deacetylases (Donmez, 2012; Duan, 2013). To date, SIRT1 has been extensively investigated due to its initial connection with lifespan extension and involvement in important biological processes. SIRT1 is protective in cell culture and animal models of PD, as long as SIRT1 deacetylates heat shock factor 1 (HSF1), peroxisome proliferators activated receptor gamma co-activator 1 alpha (PGC1α) and affects α-synuclein toxicity (Herskovits and Guarente, 2014). Unlike SIRT1, SIRT2 is present primarily in the cytoplasm, co-localizes with microtubules and deacetylates the major component of microtubules, α-tubulin at lysine 40 (North et al., 2003). In addition, SIRT2 deacetylates forkhead transcription factors of class O (FOXO) (Wang et al., 2007; Pais et al., 2013; Akbulut et al., 2015) and NF-κB (Li et al., 2013), as results of affecting apoptosis and inflammation. Moreover, sirtuin 2 is reported to exacerbate alpha-synuclein toxicity in models of Parkinson’s disease (de Oliveira et al., 2017). Consistent with the previous finding, we observe SIRT2 protein expression is highly increased in PD model. Meanwhile, SIRT1 protein expression is obviously decreased. Although SIRT2 inhibitor has been verified to play neuroprotective effects on PD (Outeiro et al., 2007; Li et al., 2013; Chen et al., 2015), these inhibitors lacked the desired isotype selectivity (Rumpf et al., 2015). Future work will be necessary to identify more potent and selective inhibitors/activators of SIRTs for neurodegeneration and may provide avenues for therapeutic intervention.

Recent studies have provided important insights into the autophagy underlying PD. It is reported that α-synuclein is predominantly degraded by the lysosomal pathways, particularly via chaperone-mediated autophagy (CMA) (Vogiatzi et al., 2008). In addition, autophagy progress regulates PD-related proteins like, such as LRRK2, PINK1, Parkin, and ATP13A2 (Martinez-Vicente, 2015). Furthermore, blocking autophagy could also trigger apoptosis (Venderova and Park, 2012). Thus, impaired autophagy causes accumulation of pernicious PD-related proteins and loss of neuron cells. In this study, we observe that p53 is highly increased in PD experimental models. As well known, p53 activates autophagy via damage-regulated autophagy modulator (DRAM) as a transcriptional target (Crighton et al., 2006). However, p53 also inhibits this process depending on its subcellular localization (Tasdemir et al., 2008) Acetylation of p53 exerts stability and transcription factor activity, which is regulated by SIRTs. Recent study reported that SIRT1 inhibited dopaminergic neurons injury via p53-caspase-3-dependent mechanism of apoptosis (Ye et al., 2013). As mentioned before, SIRT2 plays diverse role from SIRT1 in PD. In the present study, we reveal that inhibition of SIRT2 decrease the cytoplasmic p53 expression by increasing acetylation level of p53. It is well established that cytoplasmic p53 inhibit autophagy, meanwhile, the nuclear p53 promotes apoptosis. These results indicate that p53 is required for SIRT2-mediated autophagy in DA neurons, which exerts the critical role in the pathogenesis of PD. Therefore, searching therapeutic intervention for SIRT2/p53/autophagy pathway is impending.

Emerging evidences suggest that multiple miRNAs participate in the pathology of AD and PD by targeting specially genes and exhibit the therapeutic potential in neurodegenerative diseases. Identified by microarray and verified by qPCR methods, several miRNAs are highly expressed and altered in PD, such as let-7, miR-7, miR-9, miR-29, miR-34 (Qiu et al., 2014). Here, we found that miR-212-5p decreased in PD experimental model. We further revealed miR-212-5p directly and specifically regulated SIRT2 expression. There is lack of miR-212-5p study besides a recently study reported that miR-212-5p was related to fungal infections (Dix et al., 2017). Recently, besides the potential of miRNAs as biomarkers, miRNAs are also gaining attention for their therapeutic potential with far-reaching roles in neurodegenerative diseases (Zi et al., 2015). Here, we found stereotactic injection of miR-212-5p mimics into the midbrain of mice could prevent dopaminergic neuron damage and loss. An elegant study demonstrated the therapeutic feasibility and safety of miRNA in a primate disease model. The locked-nucleic-acids-modified oligonucleotides targeting miR-122 (SPC3649) method is currently being used in Phase I clinical trials for hepatitis C virus infection, probably becoming the first miRNA therapeutic target in humans (Lanford et al., 2010). However, miRNA-based therapies pose many unsolved challenges, including the specificity of miRNA function, the specific non-invasive delivery to the central nervous system, and evaluate the toxicity of therapeutic small oligonucleotides.

**FIGURE 8:**
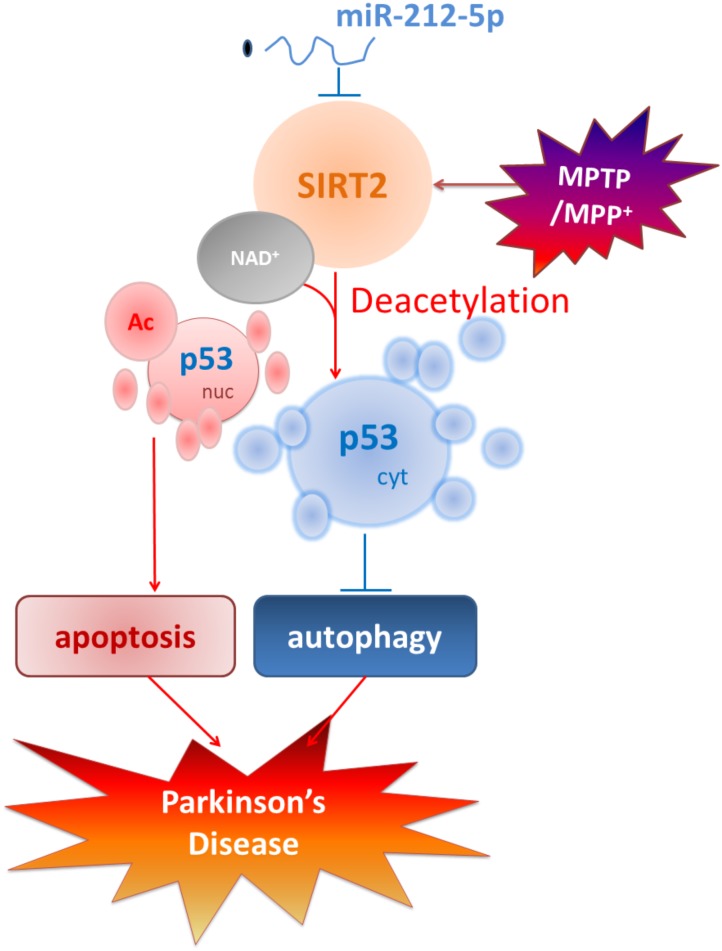
Schematic illustration demonstrates that miR-212-5p prevents dopaminergic neuron death in the pathogenesis of PD by inhibiting SIRT2. As we find, miR-212-5p is downregulated and specifically regulate the translation of SIRT2 in the midbrain or SH-SY5Y cells of PD experimental models. Furthermore, inhibition of SIRT2 promotes autophagy by decreases cytoplasmic p53 expression. According to p53 is a key deacetylation substrate of SIRT2 and plays a vital role in programmed cell death, regulation of p53 dual function by post-transcriptional regulation of SIRT2 is a potential therapy in PD.

Taken together, as shown in **Figure 8**, we demonstrate that the miR-212-5p is downregulated and specifically regulate the translation of SIRT2 in the midbrain or SH-SY5Y cells of PD experimental models. Furthermore, inhibition of SIRT2 promotes autophagy by decreases cytoplasmic p53 expression, according to the deacetylation of p53 by SIRT2. Most notably, stereotactic injection of miR-212-5p mimics into midbrain significantly improves the impairment of dopaminergic neuron by targeting SIRT2. Our study provides a direct link between SIRT2 suppression and MPTP-induced neurodegeneration. These findings will give us an insight into the potential of miR-212-5p in opening up novel therapeutic avenues for PD.

## Ethics Statement

Animal welfare and experimental procedures in this study were performed in accordance with the Guide for the Care and Use of Laboratory Animals (National Institutes of Health, the United States) and the related ethical regulations of Nanjing Medical University.

## Author Contributions

GH and SS designed the research. XH and SS performed co-transfections for the Western blotting assays, neuropathological study, RT-PCR and drafted the manuscript. XL and MJ performed the immunohistochemistry study and statistical analysis. XL and QS helped to perform the Western blotting assays and contributed materials and analysis tools. JD and ML discussed the project and gave valuable suggestions to this project. All authors read and approved the final manuscript.

## Conflict of Interest Statement

The authors declare that the research was conducted in the absence of any commercial or financial relationships that could be construed as a potential conflict of interest.

## Footnotes

1 http://www.targetscan.org/vert_72/
